# Hybrid deep learning CNN-LSTM model for forecasting direct normal irradiance: a study on solar potential in Ghardaia, Algeria

**DOI:** 10.1038/s41598-025-94239-z

**Published:** 2025-05-02

**Authors:** Boumediene Ladjal, Mohamed Nadour, Mohcene Bechouat, Nadji Hadroug, Moussa Sedraoui, Abdelaziz Rabehi, Mawloud Guermoui, Takele Ferede Agajie

**Affiliations:** 1 Department of Automation and Electromechanics, Faculty of Science and Technology, University of Ghardaïa, Ghardaïa, Algeria; 2 Applied Automation and Industrial Diagnostics Laboratory (LAADI), Faculty of Sciences and Technology, University of Djelfa, 17000 Djelfa, Algeria; 3 Laboratory of Inverse Problems, Modeling, Information and Systems (PI:MIS), University 8 Mai 1945, Guelma, Algeria; 4 Telecommunications and Smart Systems Laboratory, University of Djelfa, PO Box 3117, 17000 Djelfa, Algeria; 5 Centre de Développement des Energies Renouvelables, Unité de Recherche Appliquée en Energies Renouvelables, URAER, CDER, 47133 Ghardaïa, Algeria; 6 Department of Electrical and Computer Engineering, Faculty of Technology, Debre Markos University, 269 Debre Markos, Ethiopia

**Keywords:** Artificial neural networks, Convolutional neural network, Convolutional feed-forward back propagation, Deep learning, Feed-forward back propagation, Long short-term memory, Solar radiance forecasting, Energy science and technology, Engineering, Mathematics and computing, Physics

## Abstract

This paper provides an in-depth analysis and performance evaluation of four Solar Radiance (SR) prediction models. The prediction is ensured for a period ranging from a few hours to several days of the year. These models are derived from four machine learning methods, namely the Feed-forward Back Propagation (FFBP) method, Convolutional Feed-forward Back Propagation (CFBP) method, Support Vector Regression (SVR), and the hybrid deep learning (DL) method, which combines Convolutional Neural Networks and Long Short-Term Memory networks. This combination results in the CNN-LSTM model. Additionally, statistical indicators use Mean Squared Error (MSE), Root Mean Squared Error (RMSE), Mean Absolute Error (MAE), Mean Absolute Percentage Error (MAPE), and Normalized Root Mean Squared Error (nRMSE). Each indicator compares the predicted output by each model above and the actual output, pre-recorded in the experimental trial. The experimental results consistently show the power of the CNN-LSTM model compared to the remaining models in terms of accuracy and reliability. This is due to its lower error rate and higher detection coefficient (R^2^ = 0.99925).

## Introduction

In recent years, advancements in renewable energy technologies have gained global importance due to the increasing demand for sustainable and environmentally friendly energy solutions. Solar energy, as an abundant and clean resource, has attracted significant research attention, particularly in optimizing its production and integration into power grids. However, the efficiency of solar energy systems depends on the accurate forecasting of Direct Normal Irradiance (DNI), which is essential for planning energy generation, optimizing photovoltaic (PV) and concentrated solar power (CSP) systems, and ensuring grid stability. Accurate DNI prediction enables better energy management, reduces operational costs, and supports large-scale adoption of solar power^1–3^. Solar radiation forecasting is inherently complex due to its dependence on various meteorological factors such as cloud cover, atmospheric aerosols, temperature variations, and humidity levels. These factors exhibit nonlinear and dynamic behaviors, making it difficult for traditional forecasting methods to provide precise and reliable predictions. Conventional approaches, including Numerical Weather Prediction (NWP) models, statistical regression techniques, and empirical models, often fail to capture the intricate relationships between these meteorological variables. NWP models rely on physics-based simulations, which require high computational power, while statistical models assume linear dependencies, limiting their accuracy in highly variable weather conditions. In most cases, conventional statistical approaches, such as Linear Regression (LR), are inadequate for predicting solar radiation, necessitating more advanced methodologies^4–6^. This paper emphasizes the importance of accurate solar radiation (SR) forecasting, as it is essential for the effective development of renewable energy technologies. The city of Ghardaïa, Algeria, has been chosen as the research focus due to its high solar potential and suitability for future solar energy projects. Through studies on SR forecasting, this research aims to extend predictive capabilities to other variables related to renewable energy generation. Several forecasting methods exist for solar radiation, each suited to different timescales. Long-term forecasts often rely on statistical and physical models, while short-term and very short-term forecasts require more advanced computational techniques^7,8^. Ghardaïa’s unique climate, characterized by high variability in cloud conditions, necessitates specialized forecasting models, which are a key focus of this study. Additionally, the high demand for solar energy in Algeria’s desert regions requires precise predictions, making microclimatic-level modeling using numerical meteorological data essential^9^.

To address these limitations, Artificial Intelligence (AI) and Deep Learning (DL) techniques have emerged as powerful tools for solar irradiance forecasting. Unlike traditional models, deep learning approaches can automatically learn complex patterns from large datasets, enabling improved predictive accuracy. Among the various deep learning architectures, hybrid models combining Convolutional Neural Networks (CNNs) and Long Short-Term Memory (LSTM) networks have shown superior performance in time-series forecasting tasks. CNNs specialize in spatial feature extraction, identifying patterns in meteorological inputs such as cloud movement, temperature fluctuations, and pressure variations. Meanwhile, LSTMs are designed to process sequential data, capturing long-term dependencies and trends in solar irradiance fluctuations. By integrating CNNs and LSTMs into a single hybrid framework, forecasting models can effectively analyze both spatial and temporal dependencies, resulting in more accurate DNI predictions^10–13^. Recent studies in renewable energy systems, such as microbial electrosynthesis^14^ and biohydrogen production^15^, have demonstrated the transformative potential of hybrid architectures and adaptive optimization. These advancements underscore the need for similarly innovative approaches in solar forecasting, particularly in regions like Ghardaïa with high climatic variability.

We focus on the integration of advanced neural networks and hybrid deep learning (DL) techniques. Below are the key highlights of our study, showcasing significant advancements in AI-based approaches while addressing the limitations of conventional forecasting models:

1. Advanced AI Techniques Integration: The research utilizes a combination of conventional Artificial Neural Networks (ANNs) and DL methods, specifically CNN-LSTM models, to improve solar radiation forecasting by effectively handling complex meteorological data.
2. Focus on Ghardaïa, Algeria: Centered in Ghardaïa, a region with significant solar potential, the study tailors its findings to local climatic conditions marked by variable cloud cover and atmospheric factors.
3. Hybrid CNN-LSTM Model: By combining CNNs for spatial feature extraction and LSTMs for capturing temporal dependencies, the hybrid model effectively addresses the nonlinear nature of SR data.
4. Evaluation of ANN Architectures: The study compares various ANN architectures, including Feed-Forward Back Propagation (FFBP), Convolutional FFBP (CFBP), Support Vector Regression (SVR), and the hybrid CNN-LSTM approach, using advanced training algorithms to identify the most effective techniques for solar radiation forecasting.
5. Acknowledgement of Limitations and Contributions: While demonstrating the advantages of AI-driven approaches, the research acknowledges challenges such as data scarcity and model complexity, emphasizing the importance of optimizing input parameters for accurate solar predictions and contributing to renewable energy management.

This study focuses on developing a hybrid CNN-LSTM-based DNI forecasting framework and applying it to the Ghardaïa region in Algeria, known for its high solar potential and climate variability. The region experiences significant variations in temperature, humidity, wind speed, and dust storms, all of which impact solar irradiance levels. A reliable forecasting model tailored to these conditions is crucial for optimizing solar energy production and supporting Algeria’s renewable energy initiatives.

The dataset used in this study is sourced from NASA’s Prediction of Worldwide Energy Resources (POWER) project, covering the period from 2017 to 2023, and consists of 2575 daily observations. The key meteorological parameters include DNI, maximum and minimum temperature, wind speed, specific humidity, and surface pressure. These variables play a critical role in training deep learning models to capture variations in solar radiation and improve forecasting accuracy.

To evaluate the performance of the proposed CNN-LSTM model, its predictions are compared against conventional forecasting techniques, including Feed-Forward Back Propagation (FFBP), Convolutional FFBP (CFBP), and Support Vector Regression (SVR). The models are assessed using multiple statistical performance metrics, including Mean Squared Error (MSE), Root Mean Squared Error (RMSE), Mean Absolute Error (MAE), Mean Absolute Percentage Error (MAPE), Normalized Root Mean Squared Error (nRMSE), and the coefficient of determination (R^2^).

The results demonstrate that the CNN-LSTM model significantly outperforms traditional approaches, achieving lower forecasting errors and higher accuracy, making it a promising solution for real-world solar energy management. Beyond its technical contributions, this study highlights practical applications in optimizing solar power plant operations, improving energy grid stability, and supporting long-term renewable energy policies.

The structure of the paper is section “Related works” reviews related work on AI-based solar irradiance forecasting. Section “Materials and methods” describes the methodology, including dataset preparation, preprocessing, and model architecture. Section “Results and discussions” presents experimental results and comparative performance analysis. Finally, Section “Conclusion” concludes the study and discusses potential future research directions.

## Related works

In recent years, researchers have shown considerable interest in SR forecasting, dedicating numerous studies to this field. SR is influenced by geographic characteristics of the Earth’s surface and surrounding weather conditions. Unfortunately, it is subjected to uncontrollable changes, such as seasonal and diurnal variations, city locations, and cloud abundance. Consequently, accurate estimation of SRon Earth is crucial for maximizing solar operations. SR forecasting is crucial in renewable energy systems. Accurate predictions can minimize energy costs and optimize energy production, enabling plants to contribute effectively to energy supply chains, and supporting commerce, industry, and transportation services consistently. Various types of forecasting problems, such as electric load, wind, and solar power requests, have been a focus for decades. Today, solar power is essential and increasingly contributes to the electricity mix, becoming competitive with conventional sources. Regardless of the specific applications, solar power forecasting involves system-referenced interfaces encompassing inputs as controllable factors and the proposed output results. Paper^16^ provides an overview of advancements in forecasting methods for wind and solar energy, highlighting improvements in forecast skill, the impact of blockchain technology on data transactions, evolving business models in the renewable sector, and challenges posed by integrating high levels of renewable energy into power systems. The authors in the paper^17^ explore various methodologies for energy prediction and management in the context of renewable energy and electricity consumption forecasting. Article^18^ presents a comprehensive review of solar irradiance resources and forecasting techniques, focusing on using sensor networks for accurate solar power generation predictions. The authors in the paper^19^ investigate various methods of wind power forecasting, focusing on their analysis, prediction time scales, error measurements, and accuracy improvements to enhance the efficiency of wind energy systems. ANNs have been predominantly used in SR estimation. SR patterns undergo dynamic spatial variation in any region where geographic and weather conditions are not constant over time. Consequently, ANNs emerge as a flexible transfer function, making excellent forecasts of solar data patterns. Numerous studies have used ANNs for SR forecasting in various areas, showcasing the potential of this approach. The generalization capabilities of ANNs were highlighted by authors in^20^, demonstrating their strength in handling noisy and incomplete real-world data. This capability is crucial in modeling solar radiation, as it involves intricate and variable datasets. The review of 54 papers by Eisa underscores the importance of selecting performance criteria and network architecture as foundational steps toward successful forecasting in diverse contexts. Based on this foundation, Voyant et al.^21^ investigate the predictability of solar irradiation, particularly for insular locations without weather stations. This paper presents innovative work using physical phenomena to improve ANN predictions by addressing challenges posed by non-stationarity in solar data. By incorporating extraterrestrial irradiation coefficients, they successfully improve prediction quality, showcasing ANNs’ adaptability in unique environmental conditions. In the paper^22^, the authors advance the discourse by introducing radial basis function networks for estimating SRin areas without direct measurements. Their approach includes univariate and multivariate forecasting techniques, highlighting the versatility of ANNs in different climatic contexts. The authors demonstrate that ANNs can effectively model solar radiation, emphasizing the need for selecting appropriate input parameters to optimize performance. Hoyos-Gómez et al., in a paper^23^, address the increasing demand for accurate solar irradiance forecasting due to the proliferation of solar power generation systems. They highlight the robustness of ANNs, fuzzy logic, and hybrid models in capturing systematic errors, reinforcing ANNs’ status as a leading technique in solar irradiance modeling. Their review of existing studies indicates a significant gap in research focused on tropical environments, signaling an area for future exploration. Bou Nassif et al. review various methodologies combining ANN with statistical techniques for short-term load forecasting. They note that integrating additional input parameters can enhance ANN accuracy, further supporting the notion that ANNs can be effectively tailored to specific forecasting challenges^24^. Recent advancements are reflected in^25^, which focuses on the critical role of short-term PV forecasts in energy dispatch decisions. Their study illustrates the evolution of prediction methods, emphasizing the necessity of incorporating a broader range of meteorological features alongside traditional solar irradiance data. This approach aligns with the trend of enhancing ANN models to improve forecasting precision. Nawaeb et al., in^26^, provide a comparative review of empirical and AI-based approaches to solar irradiation forecasting, stressing that ANNs are widely used across different geographical settings. Region-specific studies have also gained prominence. For instance, Benali et al.^27^ applied a hybrid CNN-LSTM model to North African solar datasets, emphasizing the importance of localized feature engineering for arid climates. Their findings validate our methodology of integrating NASA POWER data with lagged SR measurements for Ghardaïa. Additionally, Chen et al. in^28^ explored the fusion of satellite-derived cloud dynamics with ground-based sensors using CNN-LSTM and achieved sub-hourly irradiance predictions, complementing our use of NASA’s CERES/MERRA2 dataset. Their results indicate that although empirical methods remain relevant, the trend shows a tendency toward AI models more accurately, ANNs when accuracy in predictions is required. Finally, innovative ML techniques in^29^ are applied to solar energy estimation, notably the source-free domain adaptation approach. Their work highlights the potential of advanced ANN configurations, such as LSTM networks, to adapt dynamically to changing environmental conditions, thereby improving predictive performance across different regions. Recent comparative studies, such as Wang & Li^30^, have shown that hybrid CNN-LSTM models outperform transformer-based architectures for short-term forecasts due to their localized spatial feature extraction capabilities. This aligns with our model’s design, which prioritizes short-term accuracy for energy dispatch planning. Furthermore, ethical considerations in renewable energy AI are increasingly addressed through explainability frameworks. For example, Ibrahim et al. in^31^ integrated SHAP (SHapley Additive exPlanations) into solar forecasting models to interpret feature contributions, highlighting a direction we recognize as crucial for future work. Recent advancements in hybrid models have further improved solar irradiance forecasting. Zhang et al. in^32^ proposed a spatio-temporal transformer combined with LSTM, achieving state-of-the-art accuracy in arid regions like Ghardaïa. Similarly, Kumar et al. in^33^ demonstrated that attention-augmented CNN-LSTM architectures significantly improve forecasting robustness for non-stationary solar data. These studies highlight the growing emphasis on hybrid architectures for capturing both spatial and temporal dependencies, a critical requirement for regions with high climatic variability.

In this study, we will present a comparative analysis of a CNN-LSTM model against several traditional forecasting methods, including SVR and multiple configurations of Feed Forward and Convolutional Feed-forward ANNs. The performance of each model will evaluate using key metrics: MSE, RMSE, MAE, MAPE, nRMSE, and the coefficient of determination (R^2^).

## Materials and methods

This section outlines the methodology employed in this study, including dataset acquisition, preprocessing techniques, model architecture, and performance evaluation. We focus on the integration of advanced deep learning techniques, particularly CNN-LSTM models, to enhance solar radiation forecasting by leveraging both spatial feature extraction and temporal sequence learning.

### Dataset collection

The dataset used in this study is sourced from NASA’s Prediction of Worldwide Energy Resources (POWER) project, covering the period from 2017 to 2023. The study focuses on Ghardaïa, Algeria (Table 1), an area with high solar potential and significant climatic variability, making it ideal for evaluating deep learning-based solar forecasting models. The dataset consists of 2575 daily observations of meteorological parameters critical for solar irradiance forecasting (Table 2).

**Table 1:** Dataset overview and metadata.

Category	Details
Source	NASA POWER (CERES/MERRA2)
Location	Latitude: 32.4466° N, Longitude: 3.6992° E
Elevation	522.19 m
Time Period	January 1, 2017–December 31, 2023
Resolution	0.5° × 0.625° latitude/longitude

**Table 2:** Parameters and descriptions.

Variable	Description
ALLSKY_SFC_SW_DWN	All sky surface shortwave downward irradiance (kW-hr/m^2^/day)
WS2M	WS at 2 m above ground level (m/s)
T2M_MAX	Maximum temperature at 2 m above ground level (°C)
T2M_MIN	Minimum temperature at 2 m above ground level (°C)
QV2M	Specific humidity at 2 m above ground level (g/kg)
PS	Surface pressure (kPa)

### Data preprocessing

**Fig. 1:**
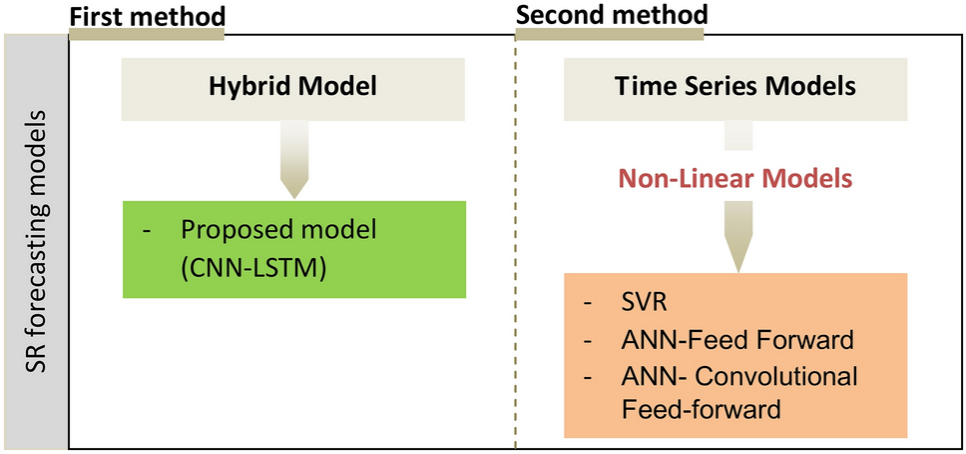
Different models for solar irradiance forecasting.

Data preprocessing is essential to ensure the quality and consistency of inputs for deep learning models. In this study, missing values are handled using linear interpolation to maintain data continuity, while all features are normalized to the [0,1] range using Min–Max normalization to enhance convergence during training. Feature engineering involves introducing temporal lag variables to help LSTMs capture time-dependent patterns and conducting correlation analysis to select the most relevant meteorological features. The dataset is then split into 85% training and 15% testing data to evaluate model performance on unseen observations. Finally, the data is formatted into 3D input sequences, making it suitable for CNN-LSTM models by incorporating multiple timesteps to capture sequential dependencies effectively. The different ML and DL models used for solar irradiance forecasting are represented in Fig. 1.

### The CNN design model

The CNN is employed to extract spatial features from meteorological data, such as temperature, humidity, and cloud cover, which are critical for understanding local weather patterns. For example, convolutional layers can identify regions of high cloud coverage that may reduce solar irradiance. The dataset, which includes climatic variables measured at 10-min intervals, is well-suited for CNN processing due to its multidimensional structure and large scale^29,34–38^.This allows the model to capture complex spatial patterns that influence solar irradiance. The architecture of CNNs typically consists of multiple layers, including convolutional layers with local receptive fields. These layers allow the model to apply convolution matrices to specific segments of the input data, mathematically represented as follows:

1 $$\left( {f*w} \right)\left( {i,j} \right) = \mathop \sum \limits_{m} \mathop \sum \limits_{n} f\left( {m,n} \right).w\left( {i - m,j - n} \right)$$

This feature extraction process not only reduces the size but also enhances the model’s resilience by maintaining invariant properties. The activation function, usually of the Rectified Linear Unit (ReLU) type, is applied post-convolution and is expressed as follows:

2 $$a\left( x \right) = {\text{max}}\left( {0,x} \right)$$

Additionally, max pooling is employed to down sample the feature maps, which can be represented as:

3 $$p\left( {i,j} \right) = \mathop {\max }\limits_{m,n \in pooling region} f\left( {i + m,j + n} \right)$$

Ultimately, the output of the fully connected layer is computed using the equation:

4 $$y = W.a + b$$

Such robustness is critical in this study, especially when considering the minimal spatial invariance of cloud patterns that influence solar irradiance. In designing the solar radiance model, the first two layers function as convolutional layers, where they capture essential features from the input data. Following these are the flatten layer, which converts the multidimensional outputs of the convolutional layers into a one-dimensional vector, allowing for seamless integration with the LSTM layers.

### The LSTM networks

**Fig. 2:**
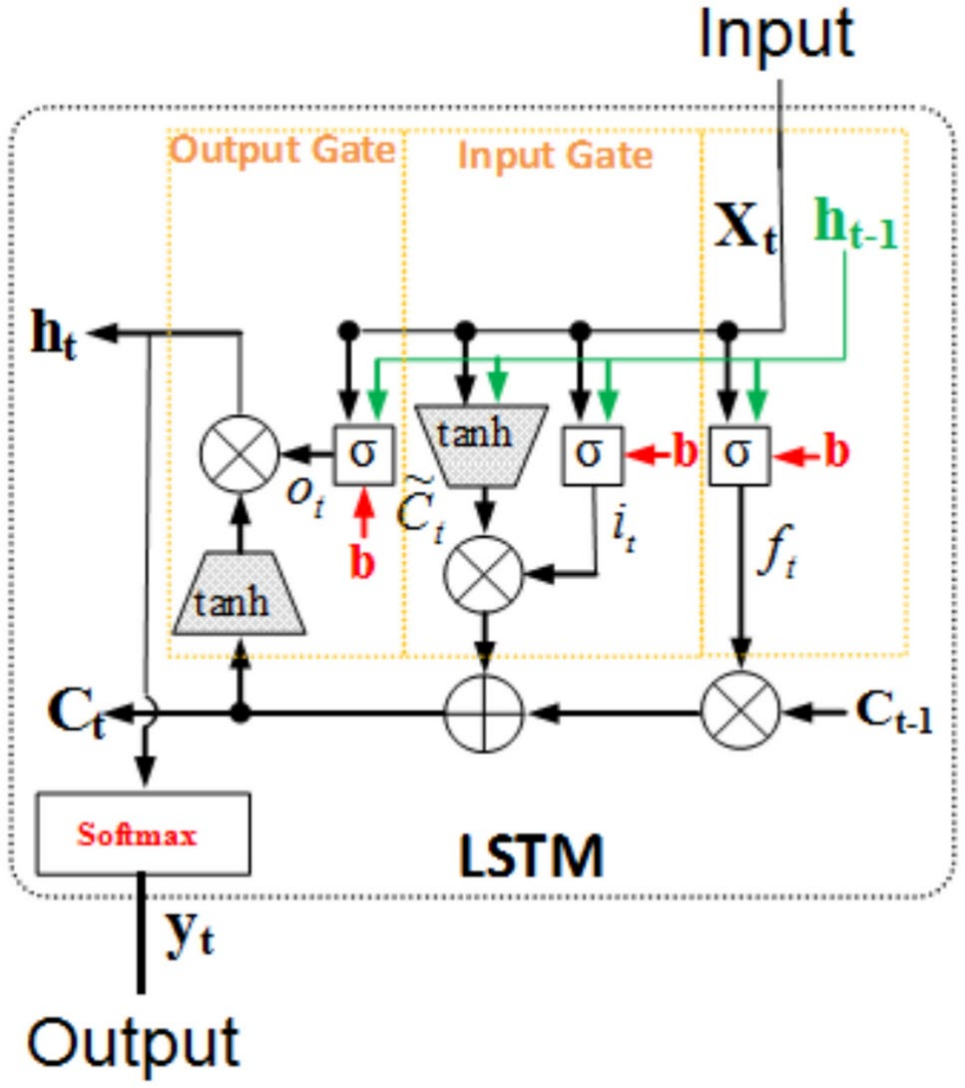
The internal structure of the LSTM cell.

The LSTM networks were first proposed in 1997 as a specific type of Recurrent Neural Networks (RNNs) designed explicitly to overcome the long-term dependency problem that architectures more general than RNNs usually suffer from. We utilize, in this study, LSTM networks in effectively forecasting direct normal irradiance by harnessing the strengths that come with LSTM in dealing with time-series data. The architecture of LSTM networks involves several types of gates, which control the flow of input data while training. This gating mechanism is relevant in our context because it allows the model to forget or remember information selectively; this is important to prevent the vanishing gradient often found in long sequences. This capability can empower LSTMs in transferring relevant information through the forecasting process, ensuring that important temporal patterns in solar irradiance data are recognized and utilized. The LSTM model utilizes three main gates to control information flow: the input gate, forget gate, and output gate. These gates are represented mathematically to decide what information should be added to or removed from the cell state. In Fig. 2 illustrate the internal structure of the LSTM cell is shown, which is used as a basic component for processing sequences in time-series data.

The input gate controls how much new information is added to the cell state and defined as:

5 $$i_{t} = \sigma \left( {W_{i} .\left[ {h_{t - 1} ,x_{t} } \right] + b_{i} } \right)$$

where $i_{t}$ represents the input gate, $W_{i}$ the weight matrix, $h_{t - 1}$ the previous hidden state, $x_{t}$ the current input, and $b_{i}$ the bias. The forget gate determines how much of the past information is retained and is given by:

6 $$f_{t} = \sigma \left( {W_{f} .\left[ {h_{t - 1} ,x_{t} } \right] + b_{f} } \right)$$

where $f_{t}$ is the forget gate, $W_{f}$ the weight matrix, and $b_{f}$ the bias term. The cell state update equation, which integrates both the forget and input gates, is expressed as:

7 $$C_{t} = f_{t} *C_{t - 1} + i_{t} *\tanh \left( {W_{c} .\left[ {h_{t - 1} ,x_{t} } \right] + b_{C} } \right)$$

where $C_{t}$ is the cell state at time *t*, $C_{t - 1}$ is the previous cell state, and $W_{c}$ the weight matrix associated with the cell state update. Finally, the output gate determines the next hidden state that is passed forward and is represented as:

8 $$o_{t} = \sigma \left( {W_{o} .\left[ {h_{t - 1} ,x_{t} } \right] + b_{o} } \right)$$

9 $$h_{t} = o_{t} *\tanh C_{t}$$

where $h_{t}$ is the new hidden state, influenced by the output of the cell state $C_{t}$, and $o_{t}$ is the output gate. By integrating LSTMs into our hybrid CNN-LSTM model, we aim to enhance the model’s ability to capture intricate temporal patterns in solar irradiance, thereby contributing to more accurate solar energy forecasting in the region of Ghardaïa, Algeria.

### The framework of the hybrid CNN-LSTM

The proposed CNN-LSTM hybrid model is designed for short-term forecasting of Direct Normal Irradiance (DNI) using meteorological data from Ghardaïa, Algeria. This framework combines the spatial feature extraction capability of CNNs with the temporal sequence learning of LSTMs, ensuring improved forecasting accuracy. CNNs identify spatial dependencies by applying convolutional filters to meteorological variables such as temperature variations, cloud coverage, and humidity distributions, which significantly influence DNI. By utilizing localized feature extraction, CNNs detect patterns such as temperature gradients preceding an increase in DNI or cloud formations reducing solar radiation, while also reducing data dimensionality and minimizing noise. The extracted spatial features are then passed to the LSTM component, which is optimized for learning long-term dependencies in solar irradiance fluctuations. LSTMs utilize memory cells and gating mechanisms to selectively retain relevant past information while discarding insignificant variations, addressing the vanishing gradient problem often encountered in deep sequence models. This allows the LSTM to recognize key temporal trends, including seasonal changes in solar radiation, daily DNI fluctuations due to sunrise and sunset, and short-term variations caused by atmospheric disturbances. The CNN-LSTM framework is trained on historical datasets, with hyperparameters optimized to minimize forecasting errors. The final model outputs precise DNI predictions, enhancing solar resource management and energy planning in arid and semi-arid regions. The design of this hybrid framework aligns with recent advancements in system optimization for renewable energy. For example,^15^ demonstrated how self-regulation and flow rate optimization enhance biohydrogen production, underscoring the value of adaptive architectures like CNN-LSTM for handling dynamic solar data. Similarly,^39,40^ emphasized the role of energy transfer mechanisms in microbial systems, which inspired our approach to capturing temporal dependencies in irradiance data. The algorithm 1 describes a CNN-LSTM model designed to forecast DNI for very short-term solar irradiance prediction.

**Figure:**
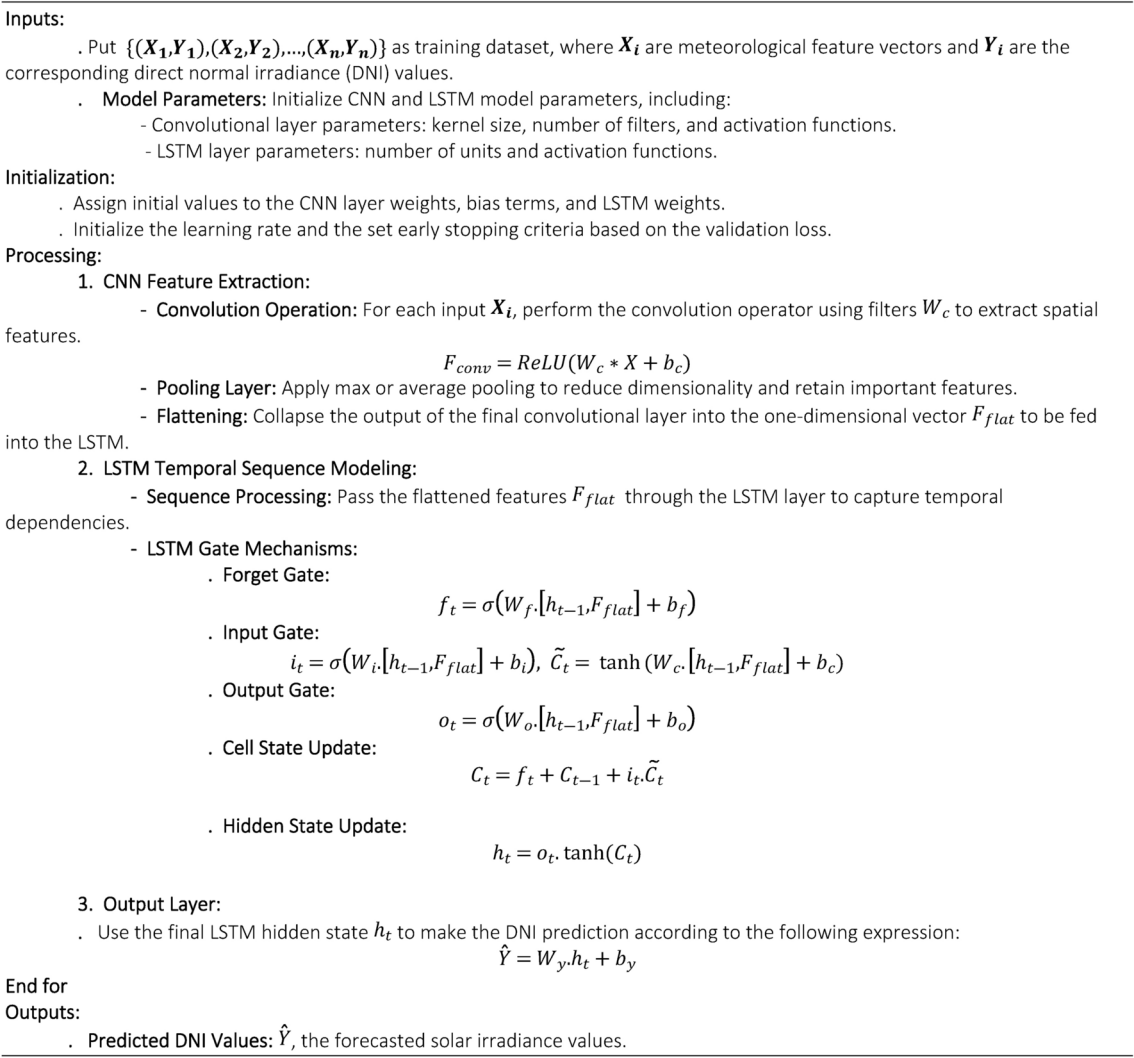
**Algorithm 1.** Algorithm corresponding the hybrid CNN-LSTM model.

### The SVR model designing

The SVR model is an effective and powerful ML technique based on the statistical learning theory introduced as a nonlinear development of support vector machines. SVM is one of the most used and effective classification and regression algorithms that perform the classification and/or regression tasks based on the principles of dimensions. It separates the two classes, or predicts numerical values, by finding the widest margin between the observations, reducing the penalty of violating the margin, and ensuring that the forecast error does not exceed an error tolerance. SVR is a non-probabilistic model, and it is included in the so-called “kernel method,” which operates directly with the input vectors in a high-dimensional implicit feature space, as a function of the distance from a prescribed center located through a kernel function by building a map of features. This space is designed to facilitate the prediction task of regression, whereas in the original feature space, the number of samples is very large. This method has shown remarkable forecasting performance, proving to be a strong generalization tool that does not need to know in advance the distribution of the error, as well as being able to easily manage small datasets with high computational efficiency. The aim is to find the hypothesis that best approximates the functional relationship between input vectors (independent variable) and dependent scalar values (response) to propose a refined forecast^34–36^.

### Design of ANNs

In general, the ANNs are computational models inspired by biological neural systems, mimicking the synaptic connections found in the human brain. These systems learn tasks or solve problems through adaptation, optimization, or experiential learning rather than following predefined rules. Similar to the human brain, ANNs represent knowledge through the weighted connections between nodes, analogous to neuronal connections. ANNs are particularly well-suited for modeling complex, non-linear relationships and are typically trained using supervised learning methods, wherein the model makes statistical inferences or decisions based on input data. By incorporating new observations, ANNs can iteratively improve their performance on specific tasks. These networks are designed to perform specific functions and can generalize across diverse types and varieties of features. Over the past decade, ANN models have demonstrated significant promise in various applications. Notably, FFBP and CFBP models have achieved high levels of success in data classification and recognition tasks. In FFBP, data flows unidirectionally from input to output through fully connected layers, with each neuron computing activations based on the weighted sum of its inputs:

10 $$a^{\left( l \right)} = f\left( {W^{\left( l \right)} a^{{\left( {l - 1} \right)}} + b^{\left( l \right)} } \right)$$

and training is performed using backpropagation, updating weights via the following expression

11 $$W^{\left( l \right)} \leftarrow W^{{\left( {l - 1} \right)}} - \eta \frac{\partial E}{{\partial W^{\left( l \right)} }}$$

Here, the CFPB enhances the FFBP by adding convolutional layers for automated feature extraction, where feature maps are computed using the following expression.

12 $$z_{i,j,k} = \mathop \sum \limits_{m,n} X_{i + m,j + n} .K_{m,n,k} + b_{k}$$

followed by pooling layers to reduce dimensions. Both architectures minimize a loss

13 $$E = \frac{1}{n}\mathop \sum \limits_{i = 1}^{N} L\left( {y_{i} ,\widehat{{y_{i} }}} \right)$$

through gradient descent, with FFBP excelling in structured data tasks and CFPB excelling in spatially hierarchical data like images.

## Results and discussions

**Fig. 3:**
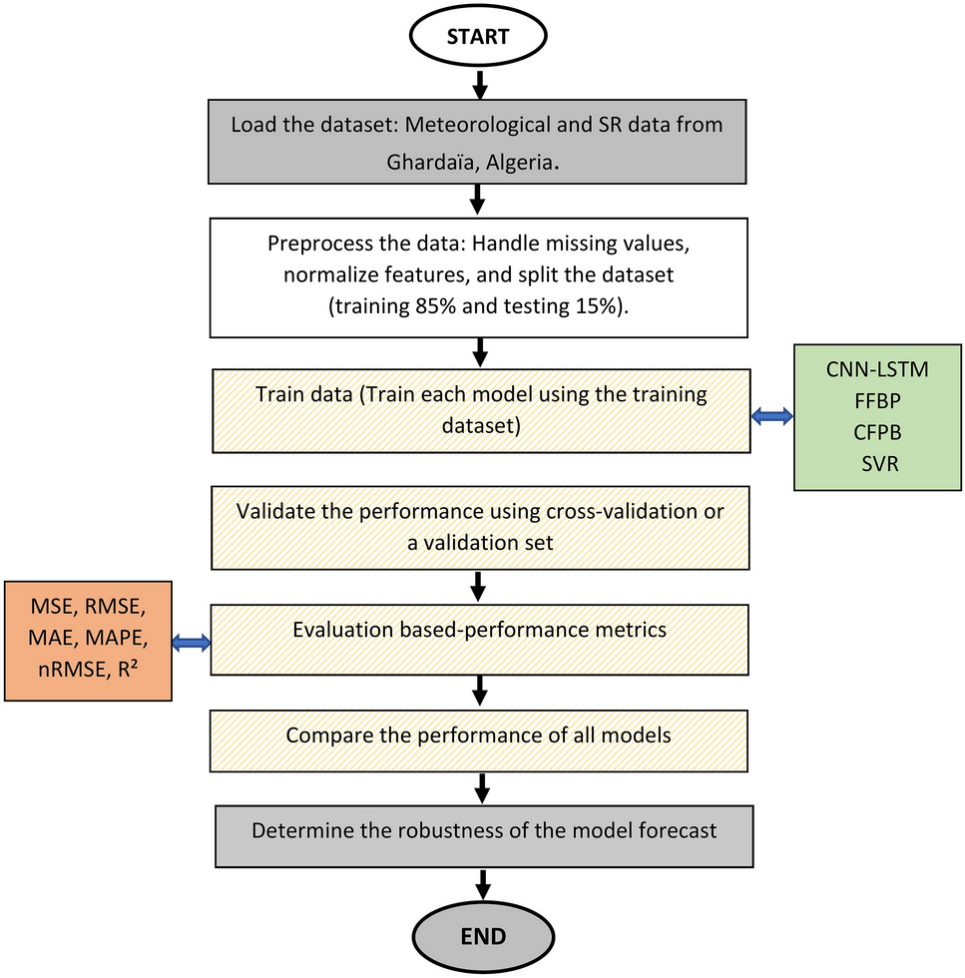
Flowchart for SR forecasting using machine learning models.

In this section, we present the performance of the proposed hybrid CNN-LSTM model for time series prediction and compare it with other models, including SVR, CFBP, and FFBP models. The performance evaluation starts with analyzing the correlation between SR and the input meteorological variables, followed by a discussion on the predictive performance of all models. Figure 3 illustrates the workflow for SR forecasting using machine learning models.

### Correlation analysis

**Fig. 4:**
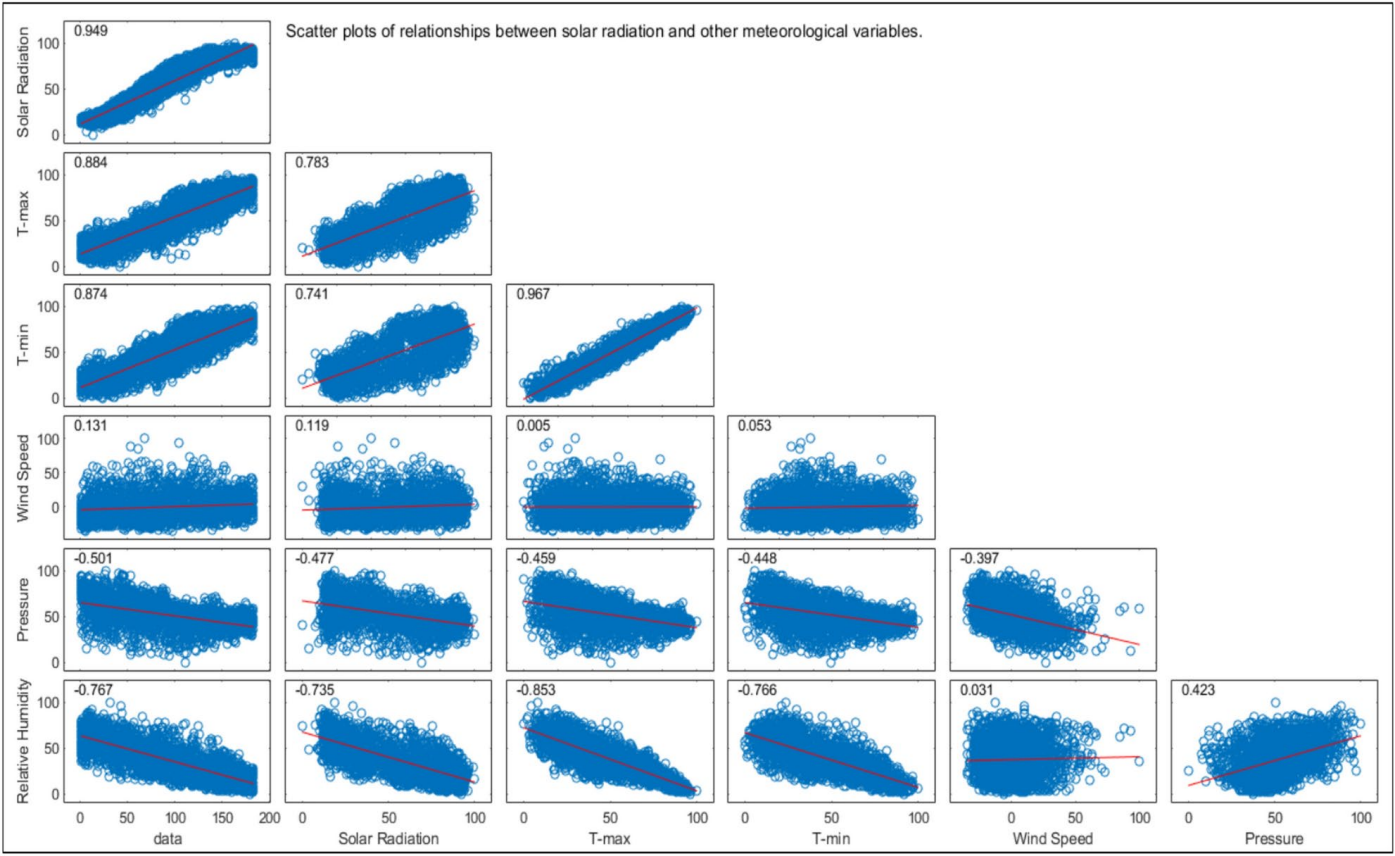
Correlation Matrix Between Solar Radiation (SR) and Meteorological Variables (Temperature, Humidity, Wind Speed) in Ghardaïa (2017–2023).

Figure 4 depicts the relationship between SR and the key meteorological variables, such as maximum temperature ($T_{max}$), minimum temperature ($T_{min}$), wind speed ($WS$), time, humidity ($Hum$), and pressure ($Pr$). According to Fig. 4, there are strong positive correlations between SR and both $T_{max}$ and $T_{min}$, indicating that higher SR values are associated with higher temperatures. Additionally, SR shows a positive correlation with time, reflecting its diurnal pattern. Conversely, SR has negative correlations with WS, Hum, and Pr, suggesting that higher SR values are linked to lower wind speeds, lower humidity, and decreased atmospheric pressure. These relationships highlight the complex interactions between SR and meteorological factors, which are essential for feature selection in predictive modeling.

**Fig. 5:**
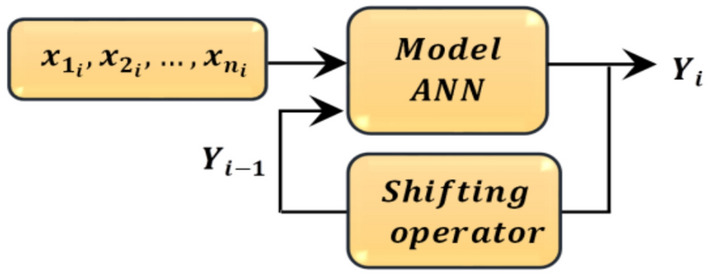
Proposed ANN architecture incorporating lagged SR and meteorological features for prediction step.

Based on the observed positive linear relationship between the current SR measurement ${\text{y}}\left( {\text{i}} \right)$ and its lagged value ${\text{y}}\left( {{\text{i}} - 1} \right)$ as shown in the scatter plots, the proposed architecture (illustrated in Fig. 5) can be expressed mathematically by

14 $${\text{y}}\left( {\text{i}} \right) = f\left( {{\text{y}}\left( {{\text{i}} - 1} \right),{\text{x}}_{1} ,{\text{x}}_{2} , \ldots ,{\text{x}}_{{\text{n}}} } \right)$$

where ${\text{y}}\left( {\text{i}} \right)$ and ${\text{y}}\left( {{\text{i}} - 1} \right)$ are, respectively, the current SR measurement and the lagged SR measurement, which presents the one-time step before occurring ${\text{y}}\left( {\text{i}} \right)$. Also, ${\text{x}}_{1} ,{\text{x}}_{2} , \ldots ,{\text{x}}_{{\text{n}}}$ are additional input features, including $T_{max}$ , $T_{min}$, $WS$, $Hum$ and pressure $Pr$. Finally, $f\left( \cdot \right)$ is the nonlinear mapping function learned by the proposed Model ANN architecture. Moreover, Eq. (14) indicates that the proposed model utilizes the temporal dependency that is captured through the lagged value ${\text{y}}\left( {{\text{i}} - 1} \right)$. It simultaneously utilizes the influence of meteorological features (${\text{x}}_{1} ,{\text{x}}_{2} , \ldots ,{\text{x}}_{{\text{n}}}$) to predict ${\text{y}}\left( {\text{i}} \right)$. Also, the nonlinear function $f\left( \cdot \right)$, implemented via the ANN model with a shifting operator, effectively captures the observed positive linear relationship and complex interactions between the inputs and the target SR values. This approach highlights the importance of incorporating both temporal dynamics and environmental variables for accurate SR forecasting. The proposed ANN Architecture incorporating lagged SR and meteorological features for prediction is shown in Fig. 5.

**Fig. 6:**
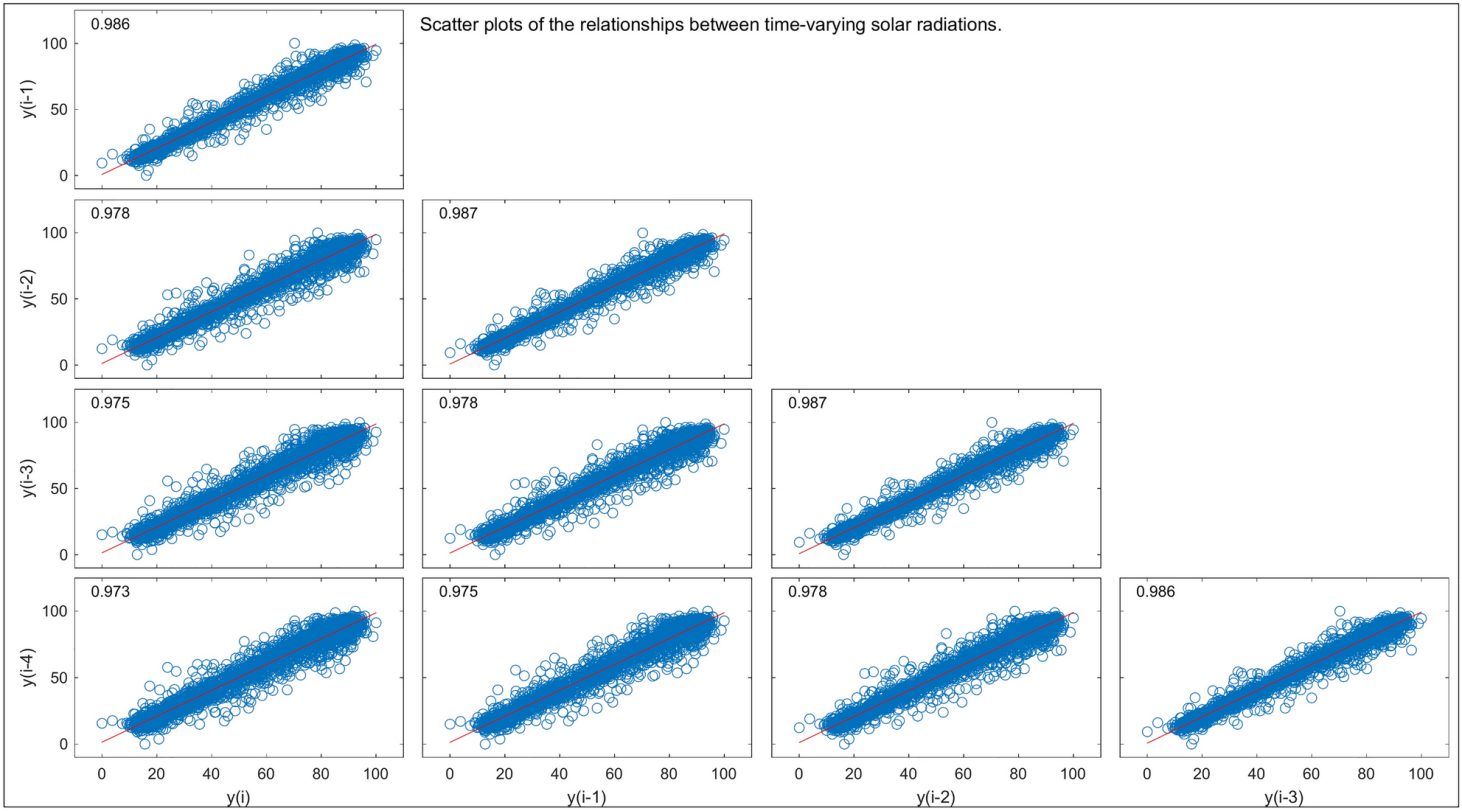
Scatter plots depicting temporal correlations between SR measurements and their lagged values.

Figure 6 gives a scatter plot collection illustrating the relationship of time-varying SR measures ${\text{y}}\left( {\text{i}} \right)$ and their lagged values ${\text{y}}\left( {{\text{i}} - 1} \right),{\text{ y}}\left( {{\text{i}} - 2} \right),{\text{ y}}\left( {{\text{i}} - 3} \right),{\text{ y}}\left( {{\text{i}} - 4} \right)$. More generally, this is known as a scatter plot matrix of the correlation coefficients. Thus, the plots of current SR measure and its past measures illustrate the temporal dependencies that may be present. The scatter plots show that there is a positive linear relationship between ${\text{y}}\left( {\text{i}} \right)$ and its lagged versions; hence, high values of current SR are usually associated with high values of past measurements. Such consistency in trends reflects the strong temporal relationship within the SR data. In addition, the coefficient of determination, ${\text{R}}^{2}$ seen in the upper-right corner of each plot represents the percentage of variance in ${\text{y}}\left( {\text{i}} \right)$ explained by each measurement. For example, the ${\text{R}}^{2}$ value of 0.986 for the relationship of ${\text{y}}\left( {\text{i}} \right)$ to ${\text{y}}\left( {{\text{i}} - 1} \right)$ indicates that 98.6% of the variability in ${\text{y}}\left( {\text{i}} \right)$ can be explained by its most recent past value. These temporal correlations and ${\text{R}}^{2}$ values underline the predictive relationships between current and lagged solar radiation, which imply that it is important to include lagged variables in time series forecasting models for an effective capture of the dynamics of SR over time.

### Model performance comparison

**Fig. 7:**
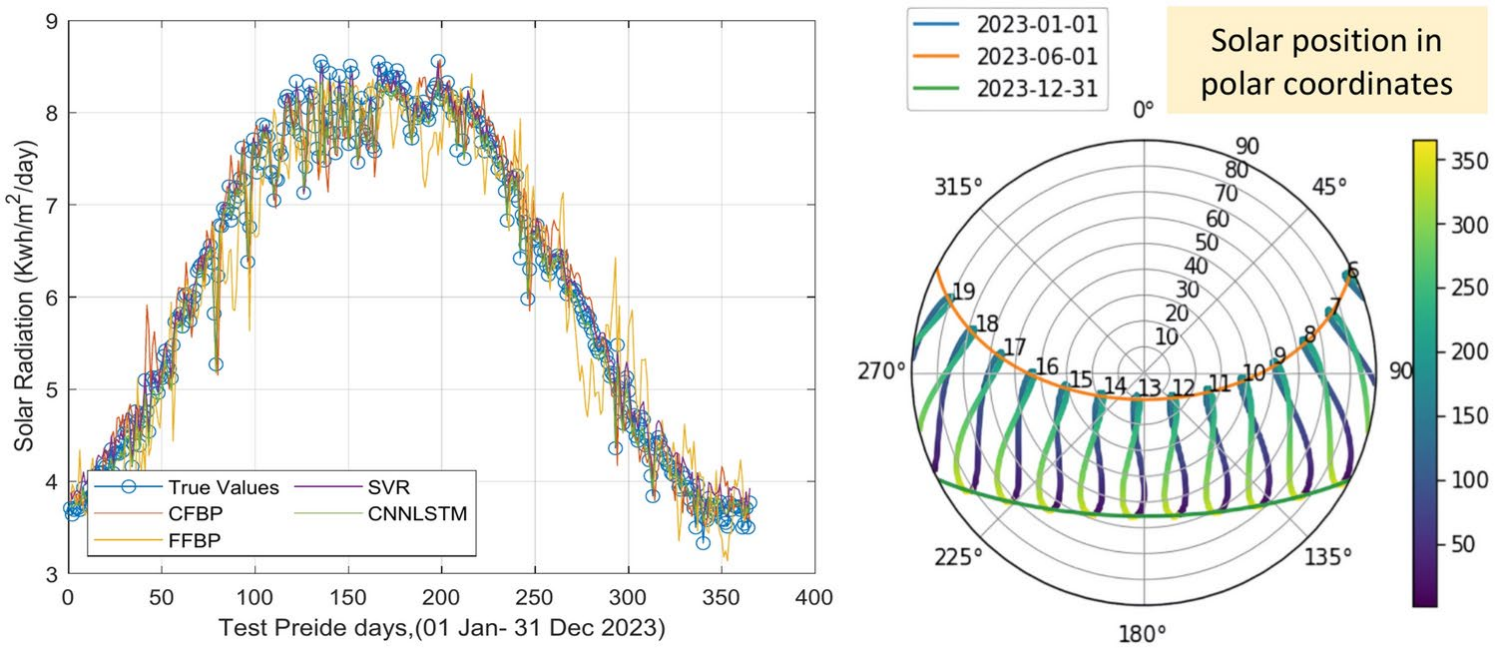
Actual vs. Predicted Solar Irradiance (SR) Values for CNN-LSTM, SVR, CFBP, and FFBP Models (2020–2023).

In this section, we compare the performance of the proposed hybrid CNN-LSTM model with other prediction models, including SVR, CFBP, and FFBP, in terms of their accuracy in forecasting SR values over a one-year period (See Fig. 7).

In Fig. 7, the performance comparison of the proposed hybrid CNN-LSTM model with the SVR, CFBP, and FFBP models is presented. The SR predictions are plotted alongside the actual observed SR measurements. This figure demonstrates that the CNN-LSTM model closely follows the observed solar irradiance trends, particularly during periods of high variability. This highlights the model’s ability to capture complex temporal dependencies, which is critical for accurate short-term forecasting. In contrast, the SVR and FFBP models show larger deviations, especially during peak solar irradiance periods.

**Fig. 8:**
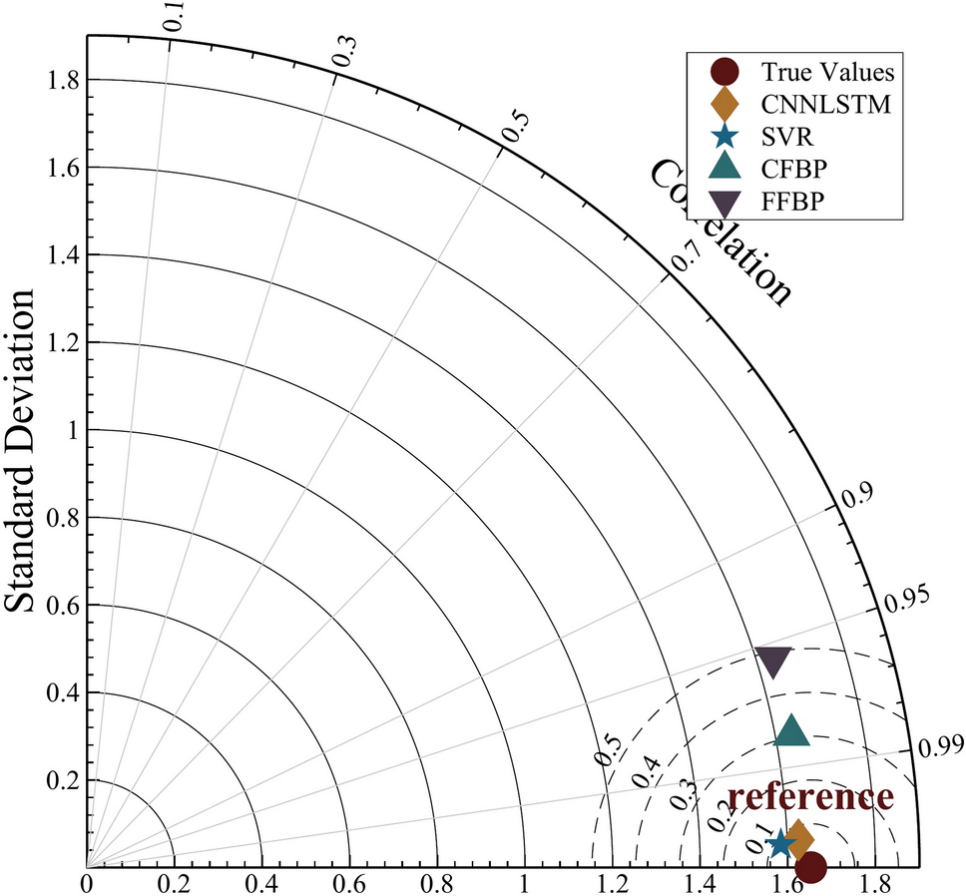
Taylor diagram comparing model performances of CNN-LSTM, SVR, CFBP and FFBP with observed SR.

Figure 8 presents a Taylor diagram comparing the predictive performance of five models: CNN-LSTM, SVR, CFBP, FFBP, and the actual SR values. The diagram evaluates the models based on three key metrics: standard deviation, correlation coefficient, and centered root mean square difference (CRMSD). The ‘True Values’ point serves as the reference, with a standard deviation and correlation coefficient of 1.0, indicating perfect agreement. The CNN-LSTM model is closest to the true values, exhibiting high correlation coefficients and low CRMSD, signifying strong agreement. In contrast, the SVR, CFBP, and FFBP models show greater discrepancies from the true values, with lower correlation coefficients and higher CRMSD. This indicates that the CNN-LSTM model significantly outperforms the others in SR prediction.

**Fig. 9:**
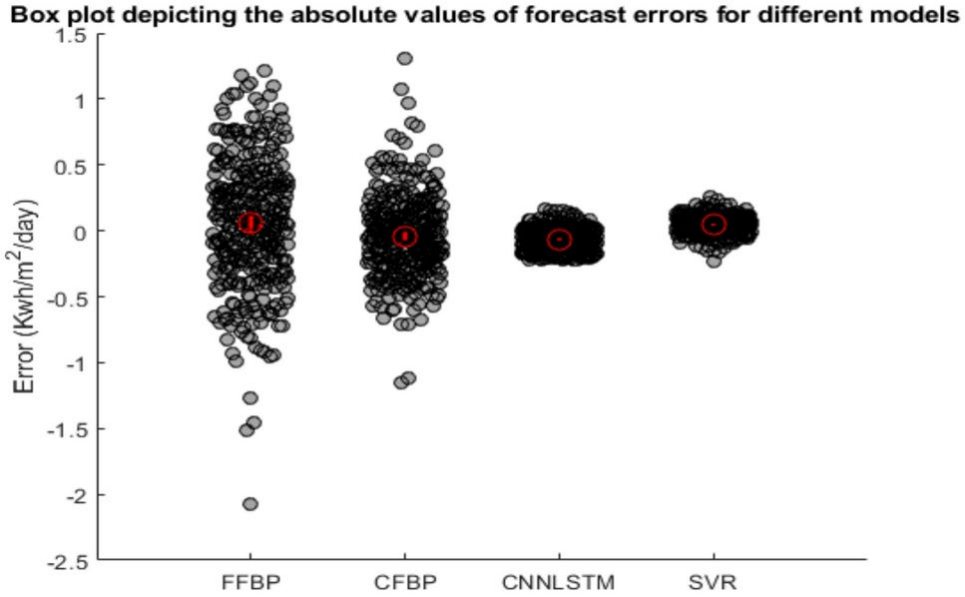
Box plot and error diagrams for FFBP, CFBP, CNN-LSTM, and SVR approaches.

Figure 9 presents a box plot comparing the absolute forecast errors of four models: FFBP, CFBP, CNN-LSTM, and SVR. The box plot displays the distribution of forecast errors, highlighting the median error, interquartile range, and minimum and maximum values for each model. The CNN-LSTM model shows the smallest median error and the narrowest interquartile range, indicating the most accurate and consistent forecasts. In comparison, the SVR model has a slightly higher median error and a wider interquartile range. The FFBP model exhibits the largest median error and the widest interquartile range, indicating higher and more variable forecast errors. The CFBP model performs better than FFBP but not as well as CNN-LSTM. Although outliers are present, indicating occasional large forecast errors for all models, the CNN-LSTM model remains the most reliable and accurate overall.

### Comparative performance evaluation using error metrics

In this section, the comparative performance of different SR prediction models is evaluated using multiple error metrics, including MSE, RMSE, MAE, MAPE, and nRMSE. These metrics are commonly used to assess the accuracy of predictive models by quantifying the discrepancies between predicted and observed values^41^. Here, the MSE calculates the average squared difference between the predicted and actual values. It is defined as:

15 $${\text{MSE}} = \frac{1}{{\text{n}}}\mathop \sum \limits_{{{\text{i}} = 1}}^{{\text{n}}} \left( {{\text{y}}_{{\text{i}}} - \widehat{{{\text{y}}_{{\text{i}}} }}} \right)^{2}$$

where ${\text{y}}_{{\text{i}}}$ is the actual value, $\widehat{{{\text{y}}_{{\text{i}}} }}$ is the predicted value, and n is the number of observations. Also, the RMSE presents the square root of the MSE. It provides an error value in the same units as the predicted data, making it easier to interpret. It is defined as^42–44^:

16 $${\text{RMSE}} = \sqrt {\frac{1}{{\text{n}}}\mathop \sum \limits_{{{\text{i}} = 1}}^{{\text{n}}} \left( {{\text{y}}_{{\text{i}}} - \widehat{{{\text{y}}_{{\text{i}}} }}} \right)^{2} }$$

Moreover, the MAE presents the average of the absolute differences between the actual and predicted values. It is particularly useful when all errors are treated equally. It is defined as:

17 $${\text{MAE}} = \frac{1}{{\text{n}}}\mathop \sum \limits_{{{\text{i}} = 1}}^{{\text{n}}} \left| {{\text{y}}_{{\text{i}}} - \widehat{{{\text{y}}_{{\text{i}}} }}} \right|$$

Similarly, the MAPE calculates the percentage error between predicted and actual values. It is often used to express the accuracy of a model in terms of percentage. It is expressed by^45,46^:

18 $${\text{MAPE}} = \frac{1}{{\text{n}}}\mathop \sum \limits_{{{\text{i}} = 1}}^{{\text{n}}} \left| {\frac{{{\text{y}}_{{\text{i}}} - \widehat{{{\text{y}}_{{\text{i}}} }}}}{{{\text{y}}_{{\text{i}}} }}} \right| \times 100$$

Finally, the nRMSE normalizes the RMSE by dividing it by the range or mean of the observed values, making it scale-independent. It is defined as^47,48^:

19 $${\text{nRMSE}} = \frac{{{\text{RMSE}}}}{{{\text{Range or Mean of y}}_{{\text{i}}} }}$$

These metrics provide different perspectives on model performance. MSE and RMSE penalize larger errors more heavily, whereas MAE treats all errors equally. MAPE is useful for understanding the error in percentage terms, and nRMSE offers a normalized error value that accounts for the scale of the data^48,48–52^. The use of these metrics helps in selecting the most appropriate model for SR prediction based on the specific characteristics of the data and the desired application. The table presents a comparative performance analysis of four SR prediction models: FFBP, CFBP, SVR, and CNN-LSTM across five error metrics: MSE, RMSE, MAPE, MAE, and nRMSE^53^.

The CNN-LSTM model consistently outperforms the other models, achieving the lowest values in all metrics, indicating its superior predictive accuracy. Specifically, it has the smallest MSE (0.0069), RMSE (0.0833), MAPE (1.1807), MAE (0.0679), and nRMSE (0.0135). These results are significantly lower than values reported in recent studies. For example, Ahmed et al.^6^ reported an MSE of 0.012 for hybrid models, and Kumari and Toshniwal^5^ achieved an RMSE of 0.15 for standalone LSTM architectures. The superior performance of the CNN-LSTM model can be attributed to its ability to capture both spatial and temporal dependencies in solar irradiance data, a feature not fully exploited by standalone or less integrated architectures.

The MSE calculates the average squared difference between the predicted and actual values, penalizing larger errors more heavily. The RMSE, derived as the square root of MSE, provides an error value in the same units as the predicted data, facilitating interpretation. The MAE, which averages absolute differences, treats all errors equally, while the MAPE expresses errors as percentages, offering intuitive insights into model accuracy. Finally, the nRMSE normalizes RMSE by the data range, enabling scale-independent comparisons.

As shown in Table 3, the CNN-LSTM model’s MSE (0.0069) is 42.5% lower than the hybrid models reported by Ahmed et al.^6^, and its RMSE (0.0833) is 44.5% lower than the standalone LSTM results from Kumari and Toshniwal^5^. These improvements highlight the effectiveness of combining CNNs for spatial feature extraction (e.g., cloud cover patterns) with LSTMs for temporal modeling (e.g., diurnal cycles). In contrast, the FFBP model exhibits the highest error values across all metrics (MSE = 0.2403, RMSE = 0.4902), reflecting its inability to handle the non-linear and dynamic nature of solar irradiance data. The SVR and CFBP models show intermediate performance, with SVR achieving an RMSE of 0.1073, which, while competitive, still lags behind the CNN-LSTM.

**Table 3:** Error Metrics for CNN-LSTM, SVR, CFBP, and FFBP Models.

	FFBP	CFBP	SVR	CCN-LSTM
MSE	0.2403	0.0957	0.0115	0.0069
RMSE	0.4902	0.3093	0.1073	0.0833
MAPE	6.7293	4.0022	1.7752	1.1807
MAE	0.3865	0.2355	0.0862	0.0679
nRMSE	0.0793	0.0501	0.0174	0.0135

**Fig. 10:**
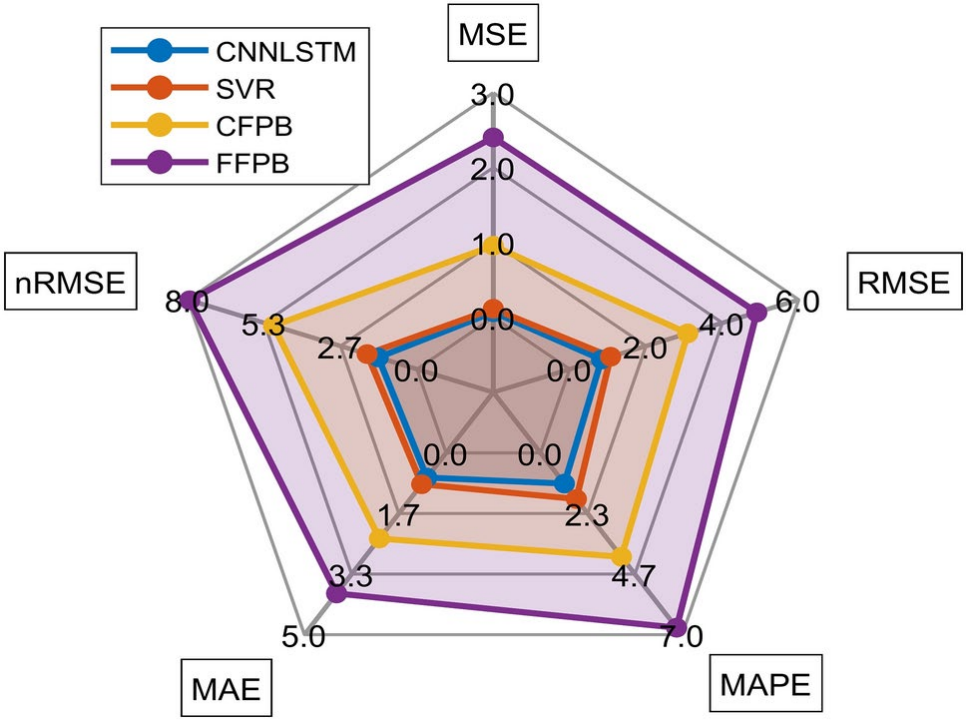
Comparison of prediction errors for different SR models based on various metrics.

The radar chart in Fig. 10 and Taylor diagram in Fig. 8 further corroborate these findings. The CNN-LSTM model’s proximity to the “True Values” reference in the Taylor diagram (high correlation, low CRMSD) underscores its alignment with observed data. Additionally, the narrow interquartile range in the box plot (Fig. 9) confirms the model’s consistency, with minimal outliers compared to FFBP and CFBP. These results demonstrate that the CNN-LSTM’s hybrid architecture is uniquely suited for solar irradiance forecasting in regions like Ghardaïa, where both spatial variability (e.g., sudden cloud cover) and temporal trends (e.g., seasonal changes) significantly impact solar potential.

**Fig. 11:**
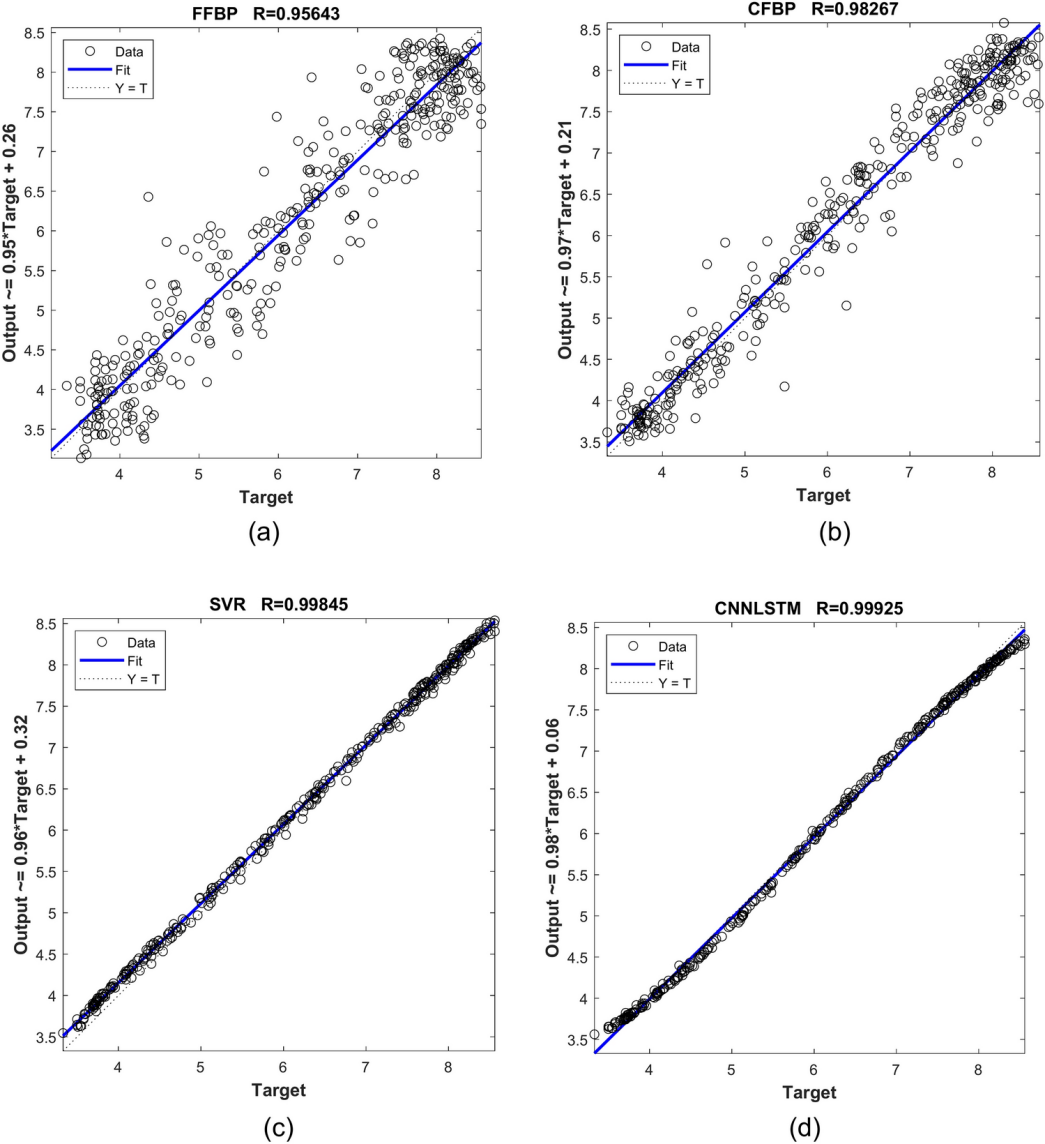
Radar chart comparison of model performance metrics. (**a**) FFBP Model; (**b**) CFPB Model; (**c**) SVR Model and (**d**) CNN-LSTM Model.

Figure 11 presents a radar chart comparing the performance of four models: CNN-LSTM, SVR, CFPB, and FFPB across five evaluation metrics: MSE, RMSE, MAPE, MAE, and nRMSE. The CNN-LSTM model consistently outperforms the others, achieving the lowest values across all metrics, indicating superior accuracy and reliability. The SVR model shows comparable but slightly inferior performance, while the CFPB model exhibits moderate results. In contrast, the FFPB model performs the worst, with the highest error values in all metrics. This comparison highlights the robustness of the CNN-LSTM model for the given task

.

Table 4 presents the coefficient of determination (R^2^) values for four models: FFBP, CFPB, SVR, and CNN-LSTM. The R^2^ values highlight the predictive accuracy of each model, with higher values indicating better performance. The FFBP model shows a reasonable fit with R^2^ = 0.95643, while the CFPB model improves to R^2^ = 0.98267, reflecting stronger accuracy. The SVR model achieves near-perfect performance with R^2^ = 0.99845, and the CNN-LSTM model outperforms all others with R^2^ = 0.99925, indicating an almost perfect correlation between predicted and actual values. These results confirm the superiority of the CNN-LSTM and SVR models for this predictive task.

**Table 4:** Coefficient of determination (R2) for model performance comparison.

Model	FFBP	CFBP	SVR	CNNLSTM
coefficient of determination R^2^	0.95643	0.98267	0.99845	0.99925

## Conclusion

This study evaluated the performance of FFBP, CFBP, SVR, and the hybrid CNN-LSTM model for solar radiation (SR) prediction using statistical metrics and visual analytics. The CNN-LSTM model outperformed all other methods, achieving the lowest errors (MSE = 0.0069, RMSE = 0.0833, MAE = 0.0679, MAPE = 1.18%, nRMSE = 0.0135) and the highest coefficient of determination (R^2^ = 0.99925), demonstrating near-perfect alignment between predicted and actual values. These results surpass recent benchmarks in hybrid models and standalone architectures, validating the effectiveness of combining spatial feature extraction (CNNs) and temporal sequencing (LSTMs). Visual tools, including radar charts, Taylor diagrams, and scatter plots, confirmed the model’s robustness in capturing complex spatial–temporal dependencies inherent in SR data. While the SVR model showed strong performance, it lagged behind the CNN-LSTM, and the FFBP/CFBP models exhibited limitations in modeling temporal dynamics.

The CNN-LSTM’s hybrid architecture positions it as a reliable tool for solar energy forecasting, particularly in regions like Ghardaïa, Algeria, where variable weather conditions challenge conventional methods. Practical applications include optimizing solar farm operations through short term forecasts for energy storage management, reducing reliance on fossil fuels during low-irradiance periods, and guiding policymakers in renewable energy planning for grid stability.

Future research should integrate additional meteorological variables such as humidity and wind speed to refine predictions and explore hybrid architectures with attention mechanisms to enhance long-term forecasting accuracy. By advancing these models, we can better support global efforts to transition toward sustainable energy systems, particularly in sun-rich regions where precise solar forecasting is critical for climate resilience and energy security.

## Supplementary Information

Supplementary Information.

## Data Availability

The data used and/or analyzed during the current study are available from co-author Dr. Abdelaziz Rabehi (rab_ehi@hotmail.fr) on reasonable request.
